# Point-of-care Ultrasound to Distinguish Subgaleal and Cephalohematoma: Case Report

**DOI:** 10.5811/cpcem.2021.3.51375

**Published:** 2021-04-19

**Authors:** Josie Acuña, Srikar Adhikari

**Affiliations:** University of Arizona, Department of Emergency Medicine, Tucson, Arizona

**Keywords:** Ultrasound, emergency medicine, pediatrics, point-of-care, case report

## Abstract

**Introduction:**

Cephalohematomas generally do not pose a significant risk to the patient and resolve spontaneously**.** Conversely, a subgaleal hematoma is a rare but more serious condition. While it may be challenging to make this diagnostic distinction based on a physical examination alone, the findings that differentiate these two conditions can be appreciated on point-of-care ultrasound (POCUS). We describe two pediatric patient cases where POCUS was used to distinguish between a subgaleal hematoma and a cephalohematoma.

**Case Reports:**

We describe one case of a 14-month-old male brought to the pediatric emergency department (PED) with concern for head injury. A POCUS examination revealed a large fluid collection that did not cross the sagittal suture. Thus, the hematoma was more consistent with a cephalohematoma and less compatible with a subgaleal hematoma. Given these findings, further emergent imaging was deferred in the PED and the patient was kept for observation. In the second case an 8-week-old male presented with suspected swelling over the right parietal region. A POCUS examination was performed, which demonstrated an extensive, simple fluid collection that extended across the suture line, making it more concerning for a subgaleal hematoma. Given the heightened suspicion for a subgaleal hematoma, the patient was admitted for further imaging and evaluation.

**Conclusion:**

Point-of-care ultrasound can be used to help differentiate between a subgaleal hematoma and a cephalohematoma to risk-stratify patients and determine the need for further imaging.

## INTRODUCTION

A cephalohematoma is a subperiosteal hematoma. It typically occurs over the parietal bones and is bound by the suture lines, meaning it cannot cross the midline. This restriction distinguishes it from a subgaleal hematoma. A subgaleal hematoma is caused by rupture of the emissary veins between the dural sinuses and scalp veins and is not bound by suture lines. Cephalohematomas generally do not pose a significant risk to the patient and resolve spontaneously.^1^ Conversely, a subgaleal hematoma is a rare but more serious condition. Because the hematoma can spread through a large plane with subgaleal hemorrhage, the amount of blood loss can be significant.^2^,^3^ It is important to distinguish between these two diagnoses as they can lead to distinct evaluation and treatment pathways. While it may be challenging to make this distinction based on a physical examination alone, the findings that differentiate these two conditions can be appreciated on point-of-care ultrasound (POCUS). We describe two cases of pediatric patients where POCUS was used to differentiate between a subgaleal hematoma and a cephalohematoma. The clinical utility of POCUS in the initial evaluation of these patients who present with an undifferentiated scalp mass are highlighted.

## CASE REPORTS

In the first case, a 14-month-old male was brought to the pediatric emergency department (PED) by his mother with concern for a head injury. The mother described that the patient was playing with his 3-year-old sister several days prior, who pushed him backward, causing him to fall from standing, hitting the right side of his head on the tile floor. She noticed a small bump on the back of his head the following day and brought the child to his pediatrician who did not feel that imaging was indicated at that time and recommended close observation at home. However, the mother described that the region continued to grow and became quite large, covering the majority of the right side of his head.

Upon initial presentation, vitals signs were as follows: temperature (tympanic) 36.8° Celsius; heart rate 156 beats per minute; respiratory rate 24 breaths per minute; blood pressure 121/88 millimeters of mercury (mm Hg); and oxygen saturation 96% on room air. On physical examination, the patient was found to be alert and interactive with no focal neurological deficits. A large, boggy area on the right parietal skull, roughly 10 centimeters (cm) in diameter, was palpated. We performed a POCUS examination to evaluate the area of concern over the patient’s head. The POCUS examination revealed a large fluid collection that did not cross the sagittal suture. Thus, the hematoma was more consistent with a cephalohematoma and less consistent with a subgaleal hematoma. Given these findings, further emergent imaging was deferred and the patient was admitted overnight for observation. The next morning, the size of the hematoma remained stable. The risks and benefits of further imaging were discussed with the mother. She opted to proceed with additional imaging given her significant concerns regarding the changes prior to arrival. A computed tomography (CT) was done, which confirmed the presence of a cephalohematoma, without any findings such as a fracture or intracranial bleed. The patient was discharged home shortly after in stable condition.

In the second case, an 8-week-old, otherwise healthy male presented to the PED with suspected swelling over the right parietal region, which was noted by the patient’s mother the day prior. The region of swelling had progressed significantly per the mother. No recent trauma was reported. The patient was born via a vaginal delivery, without forcep or vacuum assistance. A hematoma was not noted during the initial hospital stay. Presenting vitals were as follows: temperature (axillary) 36.9°C; heart rate 151 beats per minute; respiratory rate 32 breaths per minute; blood pressure 90/65 mm Hg; and oxygen saturation 96% on room air. On physical examination, the patient was alert and well-appearing. He had a normal neurological exam. There was a 7-cm boggy mass palpated on the right posterior scalp. A POCUS examination was performed, which demonstrated a large, simple fluid collection that extended across the suture line, making it more concerning for a subgaleal hematoma rather than a cephalohematoma. Given the heightened suspicion for a subgaleal hematoma, CT was performed in the PED. The CT showed a subgaleal hematoma, crossing the coronal suture posteriorly. It demonstrated normal appearance of the skull base structures with no findings of fracture or intracranial hemorrhage.

**CPC-EM Capsule**

What do we already know about this clinical entity?

*It is challenging to differentiate a subgaleal from a cephalohematoma based on physical findings alone. This diagnostic distinction is needed to guide management.*

What makes this presentation of disease reportable?

*The use of point-of-care ultrasound to distinguish between a subgaleal and a cephalohematoma in the pediatric emergency department has yet to be described in the literature.*

What is the major learning point?

*Point-of-care ultrasound can be used to help differentiate between a subgaleal hematoma and a cephalohematoma.*

How might this improve emergency medicine practice?

*Point-of-care ultrasound can be used to risk-stratify patients and assist in determining the need for further imaging.*

As no known trauma was reported, concern for a bleeding disorder or possible non-accidental trauma was considered. The patient was admitted for further evaluation so that serial head circumferences could be obtained. A skeletal survey was obtained, which showed no acute fractures or osseous abnormalities. Labs were drawn and were overall unremarkable. There were no findings to suggest a coagulopathy. Social work was also consulted. Unfortunately, during the patient’s hospital stay it was discovered that he had experienced a non-accidental trauma involving a head injury that resulted in the subgaleal hematoma. The patient had no interval increase in the size of the hematoma.

**Image 1:**
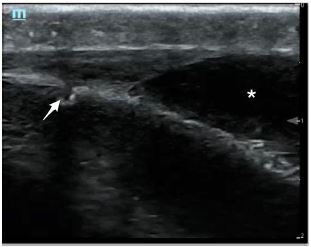
Point-of-care ultrasound demonstrating a cephalohematoma (*) that does not cross over the suture line (arrow).

**Image 2:**
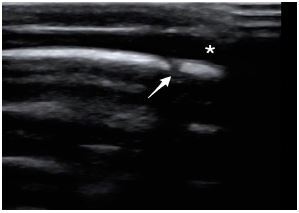
Point-of-care ultrasound demonstrating a subgaleal hematoma (*) that is seen to cross over the suture line (arrow).

A linear, high-frequency transducer was used to obtain both sets of images. The first patient had a POCUS examination that revealed an anechoic fluid collection that did not cross the sagittal suture. These findings were consistent with a cephalohematoma (Image 1). The second patient had a POCUS examination that demonstrated an anechoic fluid collection that crossed over the sagittal suture. These findings were consistent with a subgaleal hematoma (Image 2).

## DISCUSSION

**Image 3:**
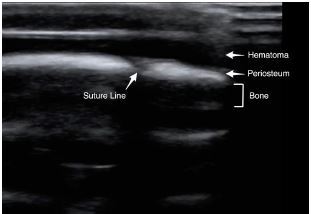
A point-of-care ultrasound demonstrating a normal cranium with an open suture line. An overlying hematoma is also identified.

The findings that differentiate a cephalohematoma from a subgaleal hematoma can be appreciated on POCUS and involve a technique easily learned by the emergency physician. A high-frequency linear transducer is used for this exam. Both images should be scanned in at least two perpendicular planes throughout the length of the hematoma to fully view the cranium below. The hematoma will typically be visualized sonographically as a superficial anechoic fluid collection. Deep to the fluid collection, the periosteum and skull are visualized as a thick line, hyperechoic to surrounding structures (Image 3).

The hematoma should be scanned throughout its entirety. While scanning through the hematoma, special attention should be made to the location of the underlying suture lines. The discontinuity is seen as a thin anechoic gap in the cranium.**4** If the fluid collection crosses over the suture line, findings are consistent with a subgaleal hematoma. If the fluid collection does not cross the suture line, results are more consistent with a cephalohematoma.

Cephalohematomas generally do not pose a significant risk to the patient and resolve spontaneously.^5^**^–7^** Conversely, a subgaleal hematoma is a rare but more serious condition. It describes bleeding in the potential space between the periosteum and the galea aponeurosis. This potential space is quite extensive, allowing bleeding to spread anteriorly to the orbital margins, posteriorly to the nuchal ridge, and laterally to the temporal fascia. Because blood is able to spread through such a large tissue plane, blood loss may be massive before hypovolemia becomes evident.^8^ Early recognition of this diagnosis is key in optimizing the outcomes for these young pediatric patients as they require careful monitoring. Patients usually require observation for frequent assessment of vital signs and head circumference measurements. A coagulopathy evaluation is often initiated as well.^8^ Although optimal imaging for subgaleal hemorrhage is by CT or magnetic resonance imaging, these studies frequently require the pediatric patient to receive some amount of sedation, or may not be readily available at the time of the patient’s presentation. Point-of-care ultrasound can be performed rapidly at the bedside and can assist in screening these patients early on, identifying those with high suspicion for subgaleal hematoma and prioritizing imaging.

## CONCLUSION

The above cases highlight the clinical utility of POCUS in the initial evaluation of pediatric patients who present with an undifferentiated scalp mass. Point-of-care ultrasound can be used to help differentiate between a subgaleal hematoma and a cephalohematoma. It is possible that these findings could assist in risk-stratifying patients and determining the need for further imaging. The approach to performing the POCUS examination is straightforward, requiring basic ultrasound technique that can be easily learned by the emergency physician.
